# Associations between paraclinical parameters, symptoms and quality of life in patients with acromegaly: a cross sectional study

**DOI:** 10.1186/s41687-022-00537-9

**Published:** 2022-12-28

**Authors:** Maximilian Cosma Gliga, Zsuzsanna Reti, Camelia Gliga, Ionela Maria Pascanu

**Affiliations:** 1 George Emil Palade University of Medicine, Pharmacy, Science and Technology of Târgu Mureș, Romania, Endocrinology Department, Targu Mures, Romania; 2 George Emil Palade University of Medicine, Pharmacy, Science and Technology of Târgu Mureș, Romania, Histology Department, Targu Mures, Romania

**Keywords:** Acromegaly, Quality of life, Symptoms, Pituitary, Patient reported outcome

## Abstract

**Introduction:**

Acromegaly is a rare chronic endocrine disorder that can lead to significant quality of life (QoL) impairment and persistent symptomatology in both biochemically uncontrolled as well as in cured or controlled patients. We aimed to conduct an observational cross-sectional study investigating the associations between biochemical disease control, associated comorbidities, and symptoms severity on QoL in a cohort of acromegalic patients.

**Methods:**

Thirty-one patients with acromegaly were enrolled in our study. AcroQoL and PASQ (Pain assessed acromegaly symptoms questionnaire) questionnaires were applied to all patients. Information about disease status, associated comorbidities, and other relevant clinical and paraclinical data were gathered.

**Results:**

Patients with uncontrolled acromegaly presented worse QoL and symptoms scores than controlled patients, but the difference was not statistically significant (AcroQoL 57.22 vs 64.04, *p* > 0.05; PASQ 12 vs 16.47, *p* > 0.05). Worse symptoms were significantly associated with impaired QoL (overall symptoms score on PASQ was negatively correlated with AcroQoL total score, *r* = − 0.61, *p* < 0.05). Cardiovascular complications were associated with lower QoL scores, but not with worse symptoms (AcroQoL total score in patients with- versus patients without cardiovascular complications: 54.89 vs 70.14, *p* < 0.05).

**Conclusions:**

Achieving biochemical control of acromegaly might not be enough to reverse the QoL impairment and improve symptomatology in acromegalic patients. While symptoms severity and the presence of cardiovascular complications seem to play an important role in reducing patients QoL, the roles of disease control, diabetes, and pituitary insufficiency are less clear.

## Background

Acromegaly is a chronic endocrine disease most commonly caused by a pituitary adenoma that produces GH (growth hormone), leading to a chronic increase of IGF-1 (Insulin Growth Factor-1). Thus, the GH-IGF-1 “axis” is considered to be the background of most pathophysiological changes that occur in acromegaly, as the increase of both these hormones causes the specific clinical and paraclinical features of the disease. Acromegaly is a rare disorder, which despite all the recent advances in therapeutic management, still evidences an unsatisfactory rate of disease control. As shown by the latest data from Acromegaly registries and large studies in European countries, the overall disease control rate ranges from over 70% in Germany and Spain, while in Romania reports estimate an unsatisfying control rate of 28.6% [1]. Acromegalic patients are known to suffer from a significant disease burden, which leads to excess mortality, morbidity, and decreased Quality of life (QoL) [2]. Both patients with biochemically active and inactive disease present with a decreased quality of life (QoL) compared to healthy populations [3]. Increased evidence indicates that medical treatment leads to an improvement of QoL in several different domains, but the relationship between the biochemical control on the GH -IGF-1 axis and QoL parameters remains unclear [4]. In recent years, many instruments for assessing QoL have been developed, some focusing on the general health-related QoL, others being disease specific [6]. Such an instrument has been developed specifically for acromegaly patients in 2006- The AcroQoL, which is currently being applied in both clinical studies, as well as in daily clinical practice. Other tools are more focused on the direct evaluation of symptoms, such as the PASQ (Pain Assessed Acromegaly Symptom Questionnaire) [7]. By the development and implementation of these questionnaires, an increasing number of physicians in modern-day medical practice are approaching clinical care from a patient-oriented method, focusing on patients’ QoL specifically.

The objective of our study was to study the associations of acromegaly with a wide spectrum of health-related quality of life aspects in a cohort of previously or newly diagnosed patients from our center. We aimed to study the relationship between the disease status of acromegaly (biochemical control on the GH-IGF-1 axis), disease duration, types of treatment options applied, as well as other clinical and paraclinical parameters on the patient-reported QoL and symptoms severity.

## Methods

We enrolled all patients that were hospitalized in our center from September 2020 until April 2021 with a diagnosis of acromegaly. All included patients underwent a full biochemical evaluation for acromegaly within the previous 3 months in accordance with our latest national protocol [8]: measurement of IGF-1 and either random GH, GH mean (the mean calculated value of multiple GH probes over 24 h), or nadir GH (lowest value of GH) during the OGTT (oral glucose tolerance test), all using standardized assays at the same laboratory. We included all patients with a clear diagnosis of acromegaly based on the national protocol criteria of diagnosis in the study. Exclusion criteria included the following: associated malignancies, acute diseases, or end-stage/severe chronic diseases unrelated to acromegaly, and psychiatric comorbidities. After signing an informed consent agreeing to be enrolled in our study, patients were requested to fill two instruments for the evaluation of QoL and symptomatology: The AcroQoL and the PASQ (Pain assessed acromegaly symptoms questionnaire). Before filling the questionnaires, a brief instructional explanation was given, ensuring patients understood how to correctly fill out both questionnaires and assured that a trained evaluator from our team was available at any time to answer questions.

The AcroQoL is a disease-specific questionnaire composed of 22 questions using a Likert scale graded from 1 to 5 for answers. The final score can be separated into 2 scales: “physical performance” and “psychological well-being”, with the latter being also divided into “appearance” and “personal relationships” subcategories. For each item, patients respond with an answer from a scale of 5 options starting from “Strongly agree” or “Always” -which is scored as 0 points, and “Never” or “Strongly disagree”, scored as 4 points. The final score, as well as the score for each category, is calculated based on a formula taking a value from 0 to 100- the lower the score, the greater the impairment on the QoL [9]. The PASQ questionnaire is based on 5 simple items where patients can rate the intensity of the main symptoms of acromegaly on a scale from 0 to 8 (0 defined as having no symptoms, higher scores reflecting more severe symptoms). Both tools used were translated and validated into the relevant language (Romanian and/or Hungarian).

We defined the patients to be biochemically controlled if having an IGF-1 value within the reference range (± 2 SD), a random GH < 1 ng/ml, a “nadir” GH during the OGTT less than 0.4 ng/ml, or GH mean < 1 ng/ml. We also collected the following data about the included patients: a sociodemographic and clinical sheet containing the following information: age, gender, body mass index (BMI), disease duration, treatments applied, current medical treatment, adenoma size at diagnosis, latest values of IGF-1 and GH, GH nadir during OGTT or GH mean during GH profiling, and the latest MRI results: tumor remnant size and the presence or absence of acromegaly specific comorbidities.

The study was approved by the Ethics Committee of the Clinical County Hospital Mures and by the Ethics Committee of “George Emil Palade” University of Medicine, Pharmacy, Science and Technology of Targu Mures (numbers 14775/10.2020 and 1167/10.2020). Statistical analysis was performed by using the GraphPad Prism version 8.0 for Windows software [10]. After applying the Kolmogorov–Smirnov test to assess the sample distribution of continuous numeric variables data, values were presented as either mean (with standard deviation) or median (with 25th and 75th percentiles). Both a Student *T*-test and Mann–Whitney test were used to assess the differences between groups of quantitative variables. Correlation coefficients were calculated by using the Spearman rank-order R. The level of statistical significance was set at a *p* < 0.05 (95% CI).

## Results

A total of 31 patients diagnosed with acromegaly fulfilling the inclusion criteria were enrolled in our study: 24 females and 7 males. The general characteristics of the included sample are presented in Table 1.

**Table 1:** General characteristics of the included sample

Total number of patients	31
Women (%)	24 (77.4%)
Men (%)	7 (22.6%)
Mean age	51.35 (± 12.5)
Mean disease duration/years since diagnosis	9.7 (± 8.97)
Surgical treatment	27 (87%)
Patients currently under medical treatment	28 (90%)
Patients who underwent radiotherapy	11 (35%)
Macroadenoma	24 (77.5%)
Microadenoma	7 (22.5%)
Mean BMI (kg/m2)	31.47 (± 7.04)

*BMI* Body mass index. For the variables expressed as means we expressed the standard deviations in parentheses

The majority of patients were harboring a macroadenoma (77.5%). Almost all patients were surgically treated in the past (87%), while 35% of patients also underwent radiotherapy. At the time of inclusion in the study, 90% of patients were receiving medical treatment for active acromegaly with either somatostatin analogues, dopamine agonists, GH-blocker (Pegvisomant), or a combination of two or three of these agents. The current biochemical control regarding the GH-IGF-1 axis and the tumor/remnant size are presented in Table 2.

**Table 2:** Current biochemical and tumoral disease status

Mean IGF-1 levels (ng/ml)	294 (± 264.8)
Uncontrolled IGF-1 levels	11 (35%)
Uncontrolled GH levels	14 (45%)
Uncontrolled biochemically (GH and/or IGF-1)	17 (54.83%)
Controlled biochemically on both GH and IGF-1	14 (45.17%)
Detectable tumor remnant on last MRI	20 (64%)

*IGF-1* Insulin growth factor-1, *GH* Growth hormone, *MRI* Magnetic resonance imagining. For the variables expressed as means we expressed the standard deviations in parentheses

64% of patients had a tumoral remnant detected at the last MRI investigation. More than half of the patients (55%) were biochemically uncontrolled with persistently active disease (IGF-1 and/or GH uncontrolled).

In Table 3 we presented the prevalence of the common acromegaly-related chronic complications.

**Table 3:** Acromegaly-related complications

Ophthalmological complications	4 (12.90%)
Cardiovascular complications	20 (64.5%)
Type 2 diabetes mellitus	12 (38.7%)
Arthropathies	11 (35.4%)
Obstructive sleep apnea	12 (38.7%)
Pituitary insufficiency	11 (35.4%)
Diabetes insipidus	2 (6.45%)
Thyroid nodules	20 (64.5%)
Thyroid cancer	2 (6.45%)

Total number of patients with various acromegaly-related complications + percentages in parentheses

The most frequent associated comorbidities were of a cardiovascular nature (64.5%). 12 patients were diabetic and 11 patients suffered from pituitary insufficiency, requiring substitutive hormonal treatment. The final means of the AcroQoL and PASQ questionnaires are presented in Table 4.

**Table 4:** AcroQol and PASQ scores

ACROQOL mean score (± SD)	
Total score	60.3 (± 20.24)
Physical	56.05 (± 22.17)
Psychological	62.73 (± 21.17)
Appearance	56.8 (± 22.61)
Interpersonal relations	68.66 (± 23.58)
*PASQ mean score (± SD)*	
Headache	2.37 (± 2.22)
Excessive sweating	3.24 (± 2.60)
Joint pain	3.41 (± 2.62)
Fatigue	3.48 (± 2.29)
Soft tissue swelling	2.31 (± 2.31)
Total score/overall	14.33 (± 8.91)

*SD* Standard deviations. AcroQoL expressed as final adjusted scores (on a scale from 0 to 100). PASQ scores expressed as means (for each symptom on a scale from 0 to 8 and the total raw score from 0 to 40)

The worst QoL, documented as the lowest AcroQoL mean score, was observed on the physical scale, while on the interpersonal relations domain patients reported the highest scores, reflecting a better QoL. Regarding the evaluation of symptoms with the PASQ scores, fatigue was the most severe symptom (3.48/8), the least severe scores being reported for headache (2.37/8).

No statistically significant correlations were found between IGF-1 values and QoL (AcroQoL) or symptoms (PASQ) (*p* > 0.05). A weak positive correlation, but statistically insignificant, was found between the IGF-1 levels and the intensity of the symptoms of headache on PASQ (Spearman *r* coefficient = 0.21). Very weak negative correlations, statistically insignificant (Spearman r coefficient between -0.1 and − 0.2) were noticed between the IGF-1 levels and all scales of the AcroQoL.

Regarding gender differences, women reported worse QoL scores on all AcroQoL scales compared to men, but the difference between the groups was not statistically significant. Men reported worse symptom scores on headache and soft tissue swelling than women (3.33 vs 2.130, 3.87 vs 2.00), but the differences were not significant for any of the symptoms.

**Fig. 1:**
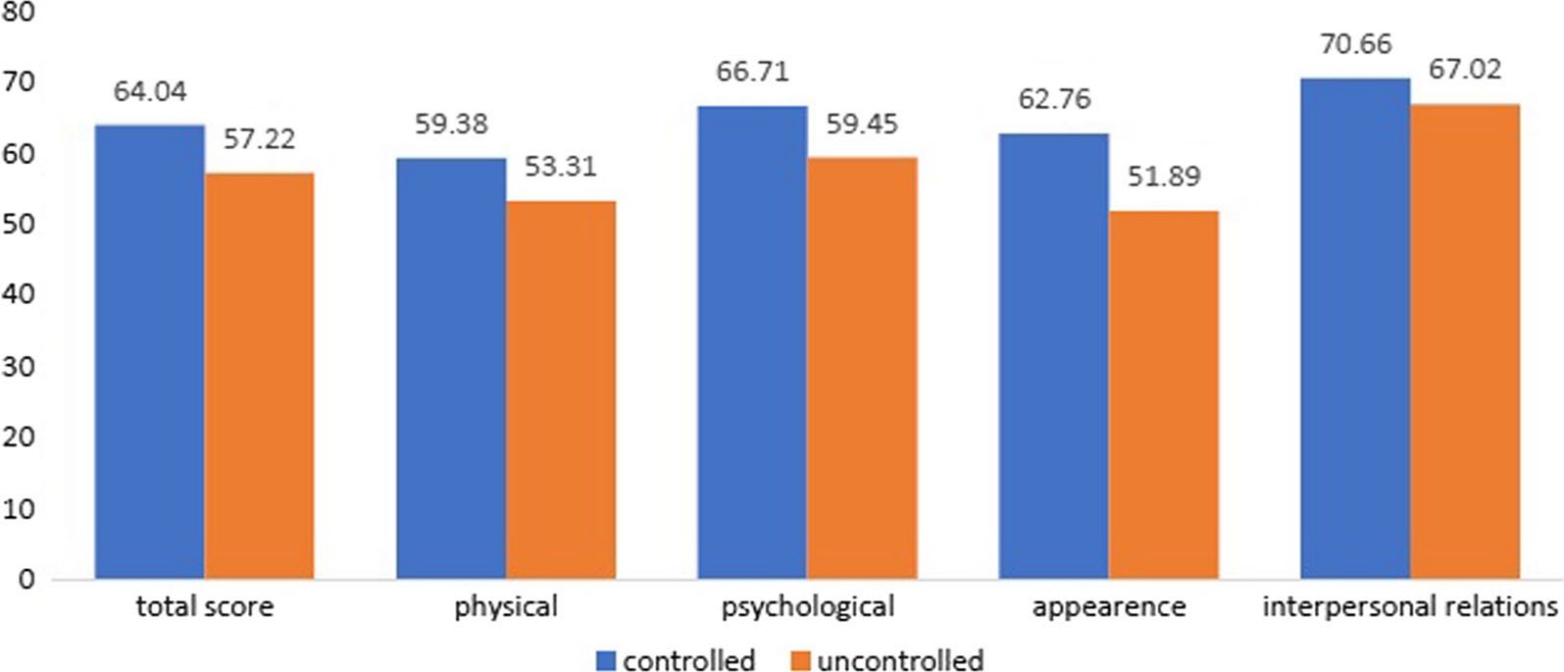
AcroQol mean scores in biochemically controlled vs biochemically uncontrolled groups

**Fig. 2:**
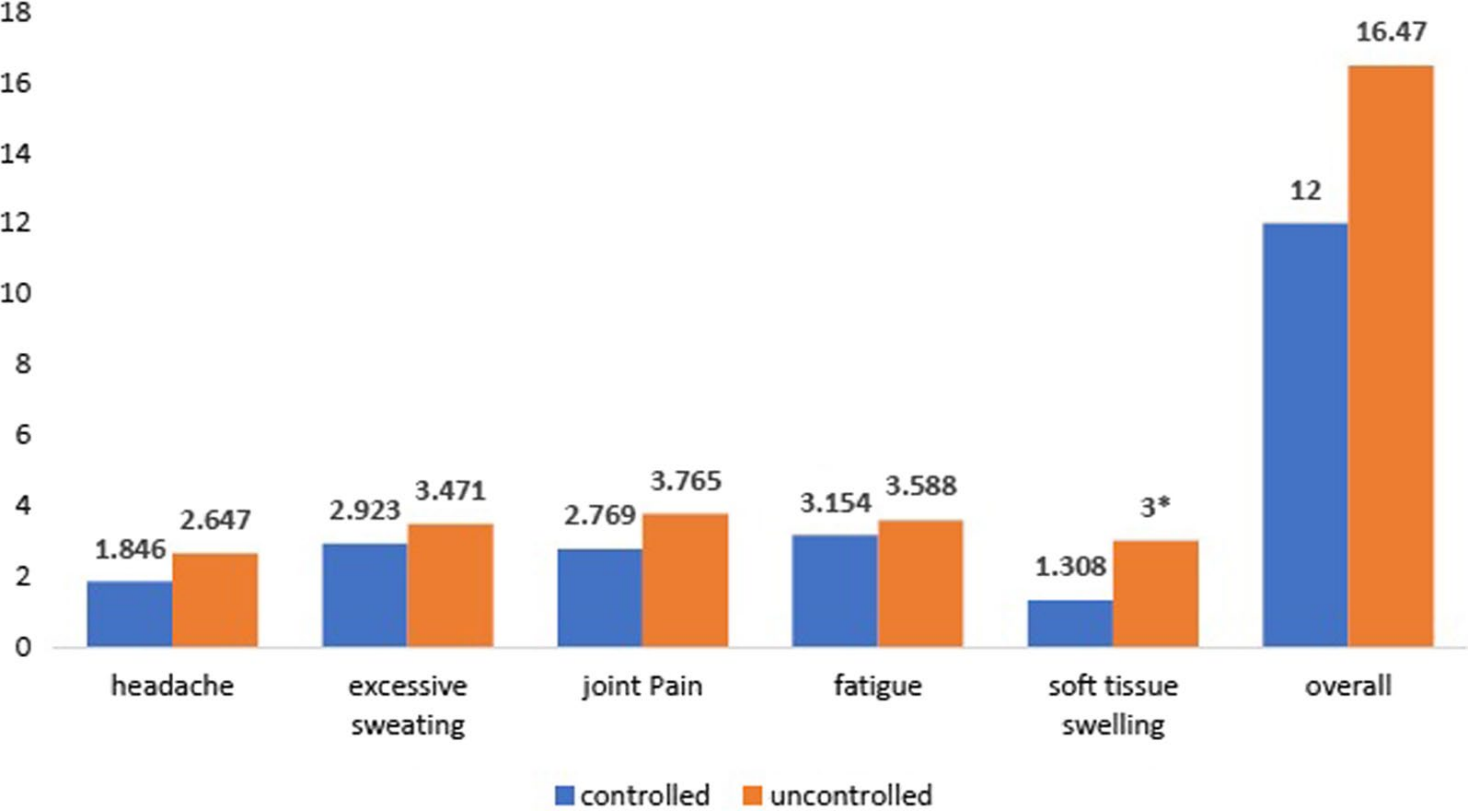
PASQ symptoms scores in biochemically controlled vs biochemically uncontrolled groups

The AcroQoL scores divided into the biochemically controlled and uncontrolled groups are represented in Fig. 1. The scores were higher on all domains in the controlled patients’ group, reflecting a better QoL, but the difference did not reach statistical significance (*p* > 0.05). Patients with uncontrolled acromegaly also reported worse symptoms compared to the controlled group, the difference being significant only for soft tissue swelling. Disease duration was weakly negatively correlated with QoL on all scales (Spearman r coefficient r between -0.15 and -0.2) but the correlation was not proven to be statistically significant. No correlations were found between disease duration symptoms severity measured by PASQ. There was a weak negative correlation between age and the AcroQoL scores on all domains, being statistically insignificant (Spearman r coefficient r between -0.11 and -0.35). The strongest negative correlation was found between the AcroQoL scores on the “Physical” dimension and age (*r* = − 0.35). The PASQ scores of the controlled and uncontrolled acromegaly groups are represented in Fig. 2. No correlation was found between age and soft tissue swelling, headaches, and excessive sweating, but there was a statistically significant and moderate positive correlation between age and symptoms severity for articular pain and fatigue (Spearman r coefficient = 0.45 and 0.53 respectively).

**Fig. 3:**
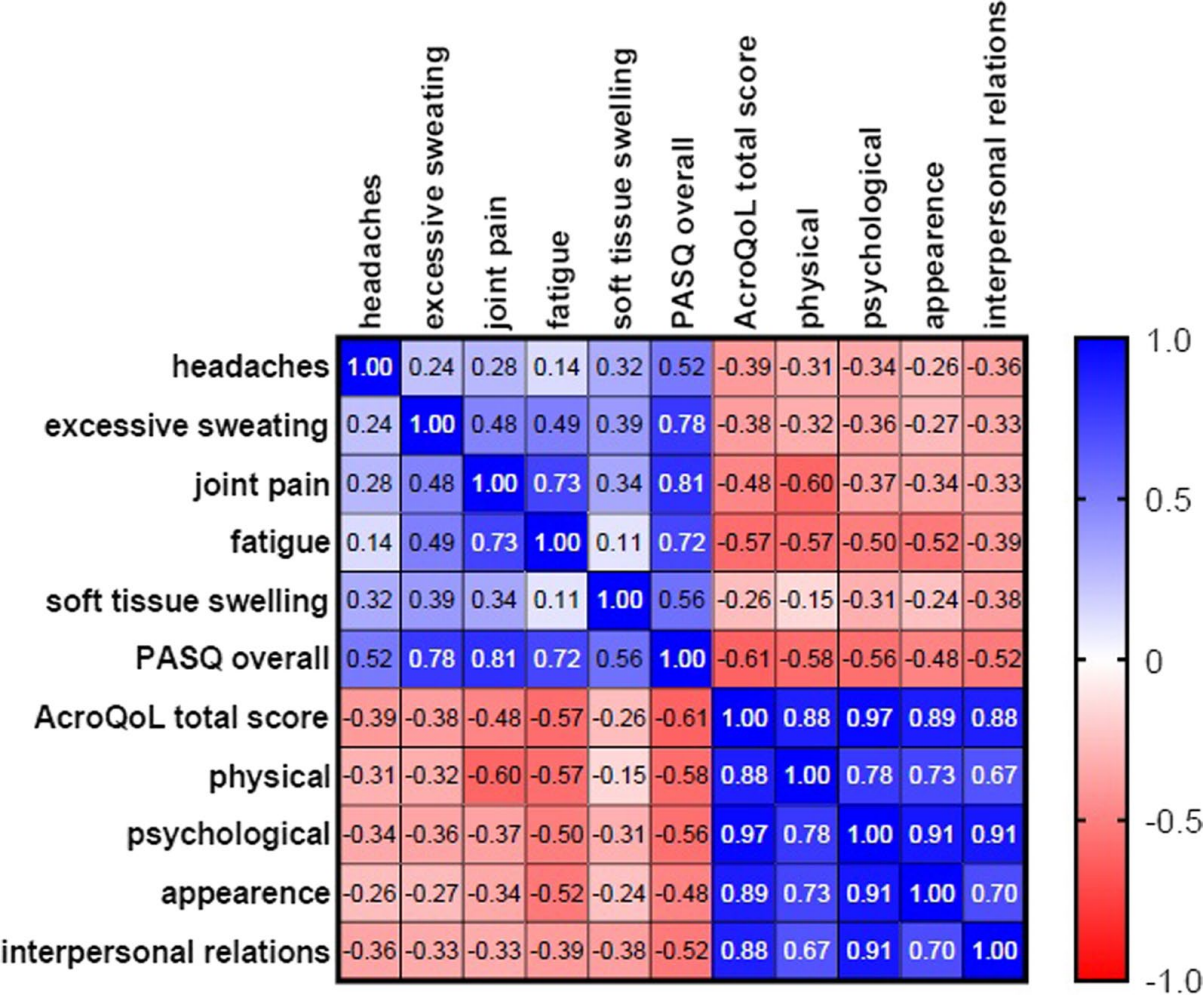
Correlation matrix between PASQ scores and AcroQol

The PASQ symptoms scores were correlated with the AcroQoL scores as presented in Fig. 3. Negative correlations with statistical significance were found between symptoms scores and lower AcroQoL: a weak correlation (*r* = − 0.31) between “headache” and “physical” dimension, a moderate to strong correlation (*r* = − 0.60) between “joint pain” and “physical,” while “joint paint” also weakly (*r* = − 0.37) correlated with the “psychological” dimension, moderate correlations between “fatigue” and all AcroQoL dimensions (r between -0.39 and -0.57), and a weak correlation (*r* = − 0.38) between “soft tissue swelling” and “interpersonal relations” dimension. Moreover, the overall symptoms score on PASQ strongly negatively correlated with the total AcroQoL score (*r* = − 0.61).

No statistically significant association was found between adenoma type (macro- vs microadenoma) or history of radiotherapy with QoL and symptoms severity. The final AcroQoL and PASQ scores in relation to the presence of the most common complications are presented in Table 5.

**Table 5:** AcroQoL and PASQ scores in relation to acromegaly complications

Cardiovascular complications	Yes, *n* = 20	No, *n* = 11	*p* value
*AcroQoL*			
Physical	51.25 (± 23.08)	64.77 (± 18.23)	0.1051
Psychological	56.96 (± 21.78)	73.21 (± 16.01)	0.0386*
Appearance	52.86 (± 21.48)	63.96 (± 23.88)	0.1957
Interpersonal relations	61.07 (± 24.88)	82.47 (± 12.92)	0.0129*
Total score	54.89 (± 20.61)	70.14 (± 16.04)	0.0425*
*PASQ*			
Headaches	2.66 (± 2.30)	1.90 (± 2.11)	0.3837
Excessive sweating	3.55 (± 2.79)	2.72 (± 2.28)	0.4151
Joint pain	3.77 (± 2.73)	2.81 (± 2.44)	0.3488
Fatigue	3.83 (± 2.43)	2.90 (± 2.02)	0.3007
Soft tissue swelling	2.61 (± 2.52)	1.81 (± 1.94)	0.3807
Overall	16.44 (± 2.23)	12.18 (± 6.60)	0.2033

Type 2 Diabetes mellitus	Yes, *n* = 12	No, *n* = 19	*p* value
*AcroQoL*			
Physical	47.14 (± 19.87)	61.68 (± 22.17)	0.0746
Psychological	54.02 (± 14.19)	68.23 (± 23.27)	0.0677
Appearance	44.64 (± 13.83)	64.47 (± 23.97)	0.0147*
Interpersonal relations	63.39 (± 18.60)	71.99 (± 26.17)	0.3309
Total score	51.52 (± 13.17)	65.85 (± 22.09)	0.0531
*PASQ*			
Headaches	3.18 (± 2.48)	1.88 (± 1.96)	0.0042*
Excessive sweating	4.63 (± 2.76)	2.38 (± 2.14)	0.0209*
Joint pain	4.18 (± 2.44)	2.94 (± 2.68)	0.2244
Fatigue	3.91 (± 1.64)	3.22 (± 2.62)	0.4439
Soft tissue swelling	3.18 (± 2.71)	1.77 (± 1.92)	0.1147
Overall	19.09 (± 8.17)	12.22 (± 8.05)	0.0354*

Pituitary insufficiency	Yes, *n* = 20	No, *n* = 11	*p* value
*AcroQoL*			
Physical	59.09 (± 14.62)	54.38 (± 25.60)	0.5797
Psychological	58.12 (± 17.83)	65.27 (± 22.84)	0.3772
Appearance	51.62 (± 19.32)	59.64 (± 24.23)	0.3535
Interpersonal relations	64.61 (± 22.22)	70.89 (± 24.56)	0.4871
Total score	58.47 (± 14.64)	61.31 (± 23.05)	0.7157
*PASQ*			
Headaches	2.33 (± 1.93)	2.36 (± 2.45)	0.9703
Excessive sweating	4.00 (± 2.34)	2.68 (± 2.58)	0.2069
Joint pain	2.44 (± 2.29)	3.78 (± 2.70)	0.2169
Fatigue	2.88 (± 1.36)	3.57 (± 2.54)	0.4551
Soft tissue swelling	2.22 (± 2.81)	2.31 (± 2.18)	0.9240
Overall	13.89 (± 7.40)	14.74 (± 9.26)	0.8122

*p* values < 0.05 were marked as bold, revealing statistical significant differences

Mean AcroQoL and PASQ scores for each scale in relation to the presence of acromegaly-specific complications. Standard deviations are expressed in parentheses. *Mann–Whitney test was applied to compare the groups (with and without complications), a *p* value < 0.05 was considered statistically significant

The presence of cardiovascular complications had a statistically significant impact on the QoL on both total scores, as well as on the psychological and interpersonal relations domains. Worse symptoms were noted in patients with cardiovascular symptoms but the differences between the groups with—and without cardiovascular complications didn’t reach statistical significance. Type 2 diabetes was associated with decreased AcroQoL scores with statistical significance on the appearance domain. Worse overall symptoms, as well as excessive sweating scores were significantly higher in the diabetic groups compared to the nondiabetic ones, as measured by the PASQ score. Pituitary insufficiency was associated with lower QoL scores on all scales but no statistically significant differences were documented.

## Discussions

While acromegaly is a rare endocrine chronic disease that causes a significant burden on patients, requiring life-long medical surveillance and sometimes multiple therapeutic strategies to achieve disease control, it is expected that besides the increased morbidity and mortality, there is also a significant quality of life impact. In our study, we evaluated the general status of QoL in acromegalic patients and the impact of biochemical status, reported symptoms, and associated comorbidities on the patient’s perceived QoL. The mean AcroQoL total score in our included sample was 60.3, which was similar to the mean score found in a large systematic review by Geraedts et al., where the mean score in cross-sectional studies from a total of 1597 patients was 62.7 [11].

The most affected QoL domains were associated with appearance and physical characteristics (mean scores: 56.8 and 56.05), indicating patients also scoring lowest on the appearance scale as compared with other studies from UK [5] and Taiwan [12]. This indicates that the facial disfigurement and bony changes that occur with long-lasting uncontrolled acromegaly represent primary determinants of decreased QoL. Regarding symptoms, fatigue and joint pain were reported as the most severe symptoms experienced by our patients through the PASQ scores (3.48 and 3.41), while headache represented the lowest severity scores (2.37), likely due to the fact that the majority of included patients were surgically treated with detectable tumor remnants present in 64% of the patients, but with dimensions stable and under 10 mm in most patients of our included sample. In comparison with the results from *Caron PJ *et al., where the total PASQ score was higher than in our patients, the symptoms severity distribution was similar, with fatigue being the most severe symptom (4.5) and headache the least severe (2.8) [13].

The main target of acromegaly treatment is reaching biochemical cure, defined by normalized IGF-1 values and a random GH < 1 ng/dl, as there is clear evidence that biochemically uncontrolled acromegaly is associated with increased morbidity and mortality. Despite recent advances in treatment,which lead to a noticeable improvement of disease control, patients with persistent active disease still reflect a 1.7–2 times higher mortality than the normal population, indicating the main causes of death being cardiovascular disease and malignancy [14, 15]. The biochemical control rate in our country is still rather unsatisfying, the largest published studies estimating a 28.6% control rate [1]. The control rate in our sample was slightly higher (45.17%), but behind the overall rate reported in the European registries, where published literature revealed a range between 56 and 76% [16–18].

While IGF-1 levels at diagnosis are considered a strong and independent negative predictor on mortality and overall morbidity in acromegalic patients [2, 15], the correlations with QoL are less established. In our study there was no significant correlation between IGF-1 levels and QoL or symptom severity, similar to observations made in a review by *Geraedts VJ *et al., where most included cross-sectional studies did not find a significant association between IGF-1 or GH levels and QoL scores implying that QoL and biochemical control are different entities, although the relationship remains unclear [11]. We found that biochemically controlled patients had higher AcroQoL scores which could have clinical significance indicating a better overall QoL compared to patients with active disease. This difference was observed on all scales, with the most notable difference being noticed on the appearance scale, yet none of the differences reached statistical significance.

Patients with active disease also scored higher on symptoms severity than controlled ones, indicating that active disease is associated with a more florid clinical picture of acromegalic patients, but the difference was significant only on soft tissue swelling. These findings could be explained by the fact that most features of acromegaly that affect QoL are secondary to chronic consequences due to the length of exposure to high IGF-1 levels rather than the simple current status of the GH-IGF-1 axis. Symptoms such as soft tissue swelling might be more clearly related to the current uncontrolled biochemical status than other symptoms or QoL aspects, the differences between disease control groups found in our study could be explained by the extracellular volume expansion and oedemas occurring in states of IGF-1 excess. Supporting this hypothesis, *Sievers C *et al. discovered in their interventional study that symptoms such as soft tissue swelling were more likely to improve after achieving biochemical control with Pegvisomant treatment compared to other symptoms such as headache, fatigue, and joint paint, where no improvement was observed, and concluded that these might be less specific to acromegaly disease control [7].

Several studies using the AcroQoL found that female gender was negatively associated with patient reported QoL,[9, 11], also being observed in the largest QoL study from our country [19]. On the other hand, *Tseng FY *et al. from the Taiwan acromegaly register indicated no significant differences between genders on QoL [12]. In our sample, women reported worse QoL than men on all AcroQoL scales but the differences did not reach statistical significance.

As expected, we found that overall symptoms severity, measured by the PASQ total score, was correlated with lower QoL. A clinically relevant finding was that bodily pain, both headache as well as neuropathic pain, was found to be a determinant of impaired QoL for acromegalic patients [4], which was observed in our study as a worsening headache association with a lower QoL score on the physical dimension of the AcroQoL, while the other areas of QoL were not significantly affected. Although fatigue is a relatively non-specific symptom that could be caused by a large spectrum of associated diseases, the symptom severity scores on PASQ were negatively correlated with all QoL dimensions, both physical and psychological. Acromegalic patients are known to suffer from higher rates of affective disorders such as depression, where fatigue might be a consequence of these psychocognitive impairments, leading to a lower perceived QoL which might be less related to the acromegalic disease control per se. Therefore, additional emphasis on patient counseling and psychiatric treatment for these conditions is necessary [20].

When it comes to adenoma type, similar to the findings by *Scânteie CL *et al. [19], patients with microadenomas scored higher than those with macroadenomas on the AcroQoL, which is expected as these patients usually have an indolent evolution and a better response to therapy, yet the difference was statistically insignificant in our lot, probably due to the low number of included patients with microadenoma. Radiotherapy is usually applied for acromegalic patients non-respondent to all other treatment modalities which have an aggressive disease evolution. Moreover, some detrimental side effects, such as neuro-cognitive dysfunction or late hypopituitarism, are not infrequent in acromegalic patients undergoing radiotherapy. It was therefore expected that a history of radiotherapy might be related to impaired QoL, which was confirmed in older studies [21, 22]. On the other hand, more recent findings including some from our country reported no significant associations between radiotherapy history and QoL [12, 19]. Our study found no differences in terms of QoL or symptoms between the previously irradiated group compared to the patients that didn’t undergo radiotherapy.

Acromegalic patients frequently suffer from cardiovascular complications, such as arterial hypertension, cardiomyopathy, and associated consequences. Besides being some of the major causes of excess mortality, these associated comorbidities may also lead to impairment in patients’ QoL. In the study by *Scânteie CL *et al. [19], it was found that cardiovascular complications played a negative role on acromegalic patients’ QoL*.* In our study we found that cardiovascular complications negatively impacted patients’ QoL and there was also a higher reported symptoms severity in this group of patients which didn’t reach statistical significance but might be due to the more aggressive and prolonged disease evolution of these patients.

Metabolic complications, such as diabetes mellitus, are frequently associated in acromegalic patients. *Webb *et al. found that diabetic acromegalic patients presented worse QoL than their non-diabetic peers [23], while *Scânteie CL *et al. found that both diabetes and cardiovascular complications impacted the QoL of acromegalic patients [19]. Diabetic patients in our sample had a worse QoL only on the appearance dimension, while the severity of symptoms was significantly higher in diabetic patients, with the most notable difference found for excessive sweating, a symptom which can also overlap with the clinical picture of uncontrolled diabetes with autonomic neuropathy.

As acromegaly is frequently diagnosed in advanced macroadenoma stages, where multiple aggressive treatment strategies are required to achieve disease control, a commonly occurring complication in these patients is hypopituitarism. In our study, we found pituitary insufficiency in 35.4% of the patients, lower than the prevalence found in another study from Romania (63.2%) [19]. Data about pituitary insufficiency and impaired QoL is conflicting, with some studies indicating a negative determinant on overall QoL [21]. More recent studies found no evidence that patients with hypopituitarism suffer from worse QoL, probably due to the fact that most patients are adequately controlled with hormone replacement for the insufficient lines [19]. In our study pituitary insufficiency was not associated with worse AcroQoL scores or higher symptoms severity.

Although acromegaly is a rare disorder, a main limitation of this study is the limited sample size (*n* = 31). Moreover, a significant limitation can be attributed to the cross-sectional design, which made it impossible to assess QoL and symptoms variability over time. Another important limitation is due to the highly variable timeframe after which patients achieved biochemical control of the GH-IGF-1 axis. Lastly, we would also mention the lack of a matched control group of non-acromegalic patients as a limitation, especially for the assessment of associated comorbidities impact on QoL and symptoms.

In conclusion, we found that both QoL and symptoms severity play an important role in the overall outcome of acromegalic patients, and instruments such as the AcroQoL and the PASQ questionnaire might be useful tools to implement in both clinical studies as well as in daily practice to improve the therapeutic management of acromegaly. There is a clear relationship between symptom severity and impaired QoL, while the relationships between biochemical control, gender, previous radiotherapy, hypopituitarism, and QoL are less evident. Cardiovascular complications appeared to be associated with lower QoL, but it remains to be elucidated how specific this is to the acromegalic population and whether biochemical control can lead to improvements.

## Data Availability

The data associated with the paper are not publicly available but are available from the corresponding author on reasonable request.

## References

[1] Niculescu DA, Baciu IF, Capatina C (2017). Acromegaly treatment in Romania. How close are we to disease control?. Endokrynol Pol.

[2] Sherlock M, Ayuk J, Tomlinson JW (2010). Mortality in patients with pituitary disease. Endocr Rev.

[3] Kyriakakis N, Lynch J, Gilbey SG (2017). Impaired quality of life in patients with treated acromegaly despite long-term biochemically stable disease: results from a 5-years prospective study. Clin Endocrinol (Oxf).

[4] Crespo I, Valassi E, Webb SM (2017). Update on quality of life in patients with acromegaly. Pituitary.

[5] Paisley AN, Rowles S, v., Roberts ME, (2007). Treatment of acromegaly improves quality of life, measured by AcroQol. Clin Endocrinol (Oxf).

[6] Brazier JE, Harper R, Jones B, NM, (1992). General practice validating the SF-36 health survey questionnaire: new outcome measure for primary care. British Med J.

[7] Sievers C, Brübach K, Saller B (2010). Change of symptoms and perceived health in acromegalic patients on pegvisomant therapy: a retrospective cohort study within the German pegvisomant observational study (GPOS). Clin Endocrinol (Oxf).

[8] Romanian national health insurance house “CNAS” official website, therapeutic protocols, https://cnas.ro/protocoale-terapeutice/, Accessed 5 Nov 2022

[9] Badia X, Webb SM, Prieto L, Lara N (2004). Acromegaly quality of life questionnaire (AcroQoL). Health Qual Life Outcomes.

[10] GraphPad Software, La Jolla California USA, www.graphpad.com".

[11] Arosio M, Reimondo G, Malchiodi E, et al (2012) Predictors of morbidity and mortality in acromegaly, an Italian survey 10.1530/EJE-12-0084 22596288

[12] Kasuki L, da Rocha P, S, Lamback EB, Gadelha MR, (2019). Determinants of morbidities and mortality in acromegaly. Arch Endocrinol Metab.

[13] Schöfl C, Franz H, Grussendorf M (2013). Long-term outcome in patients with acromegaly: analysis of 1344 patients from the German acromegaly register. Eur J Endocrinol.

[14] Howlett TA, Willis D, Walker G (2013). Control of growth hormone and IGF1 in patients with acromegaly in the UK: responses to medical treatment with somatostatin analogues and dopamine agonists. Clin Endocrinol (Oxf).

[15] Bex M, Abs R, T’Sjoen G (2007). AcroBel - the Belgian registry on acromegaly: a survey of the “real-life” outcome in 418 acromegalic subjects. Eur J Endocrinol.

[16] Geraedts VJ, Andela CD, Stalla GK (2017). Predictors of quality of life in acromegaly: no consensus on biochemical parameters. Front Endocrinol (Lausanne).

[17] Tseng FY, Chen ST, Chen JF (2019). Correlations of clinical parameters with quality of life in patients with acromegaly: Taiwan acromegaly registry. J Formos Med Assoc.

[18] Caron PJ, Bevan JS, Petersenn S (2016). Effects of lanreotide Autogel primary therapy on symptoms and quality-of-life in acromegaly: data from the PRIMARYS study. Pituitary.

[19] Scânteie CL, Leucuţa DC, Ghervan CMV (2021). Quality of life in patients with acromegaly – a romanian single center cross-sectional study. Acta Endocrinol (Copenh).

[20] Sievers C, Dimopoulou C, Pfister H (2009). Prevalence of mental disorders in acromegaly: a cross-sectional study in 81 acromegalic patients. Clin Endocrinol (Oxf).

[21] Vandeva S, Yaneva M, Natchev E (2015). Disease control and treatment modalities have impact on quality of life in acromegaly evaluated by acromegaly quality of life (AcroQoL) questionnaire. Endocrine.

[22] Biermasz NR, van Thiel SW, Pereira AM (2004). Decreased quality of life in patients with acromegaly despite long-term cure of growth hormone excess. J Clin Endocrinol Metab.

[23] Webb SM, Badia X (2016). Quality of life in acromegaly. Neuroendocrinology.

